# miR-210 Mediated Hypoxic Responses in Pancreatic Ductal Adenocarcinoma

**DOI:** 10.1021/acsomega.4c08947

**Published:** 2024-11-20

**Authors:** Maria Mortoglou, Mutian Lian, Francesc Miralles, D. Alwyn Dart, Pinar Uysal-Onganer

**Affiliations:** † Cancer Mechanisms and Biomarkers Research Group, School of Life Sciences, University of Westminster, London W1W 6UW, U.K.; ‡ School of Health and Medical Sciences, City St George’s, University of London, Cranmer Terrace, London SW17 0RE, U.K.; § UCL Cancer Institute, University College London, Paul O’Gorman Building, 72 Huntley Street, London WC1E 6DD, U.K.

## Abstract

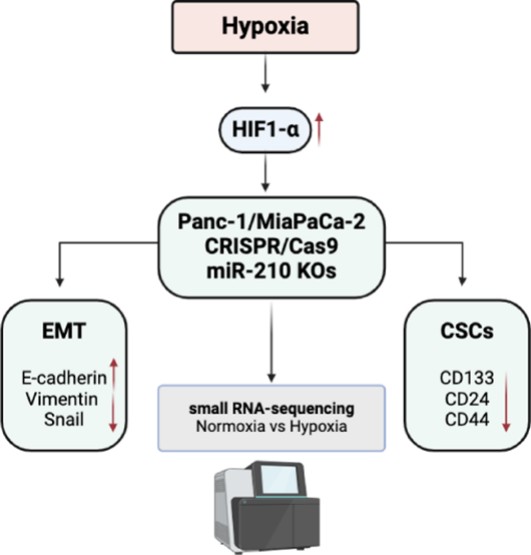

Pancreatic ductal adenocarcinoma (PDAC) is one among the most lethal malignancies due to its aggressive behavior and resistance to conventional therapies. Hypoxia significantly contributes to cancer progression and therapeutic resistance of PDAC. microRNAs (miRNAs/miRs) have emerged as critical regulators of various biological processes. miR-210 is known as the “hypoxamir” due to its prominent role in cellular responses to hypoxia. In this study, we investigated the multifaceted role of miR-210 in PDAC using miR-210 knockout (KO) cellular models to elucidate its functions under hypoxic conditions. Hypoxia-inducible factor-1α (HIF1-α), a key transcription factor activated in response to low oxygen levels, upregulates miR-210. miR-210 maintains cancer stem cell (CSC) phenotypes and promotes epithelial–mesenchymal transition (EMT), which is essential for tumor initiation, metastasis, and therapeutic resistance. Our findings demonstrate that miR-210 regulates the expression of CSC markers, such as CD24, CD44, and CD133, and EMT markers, including E-cadherin, Vimentin, and Snail. Specifically, depletion of miR-210 reversed EMT and CSC marker expression levels in hypoxic Panc-1 and MiaPaCa-2 PDAC cells. These regulatory actions facilitate a more invasive and treatment-resistant PDAC phenotype. Understanding the regulatory network involving miR-210 under hypoxic conditions may reveal new therapeutic targets for combating PDAC and improving patient outcomes. Our data suggest that miR-210 is a critical regulator of HIF1-α expression, EMT, and the stemness of PDAC cells in hypoxic environments.

## Introduction

Pancreatic ductal adenocarcinoma (PDAC) represents 90% of all pancreatic tumors, and is a highly aggressive and deadly cancer. The lack of early detection, high metastatic potential, and limited treatment options contribute to its poor prognosis.^1^ The 5-year survival rate remains alarmingly low, at less than 10%.^2^ While surgical resection and chemotherapy, including Gemcitabine and FOLFIRINOX, have slightly improved survival in early stage patients, these treatments offer limited effectiveness for those with late-stage PDAC.^3^ To date, FOLFIRINOX and Gemcitabine combined with Nab-Paclitaxel remain the most effective and widely prescribed treatment for PDAC patients.^4^ More recent studies have also suggested the use of FAK and PDK inhibitors as potential antitumor therapeutic agents against PDAC.^5,6^ Despite advances in our understanding of cancer biology and improvements in therapeutic strategies, PDAC continues to pose a significant clinical challenge, underscoring the urgent need for novel diagnostic and therapeutic approaches.

The tumor microenvironment can influence various cellular processes, including proliferation, invasion, chemosensitivity, angiogenesis, and tumorigenesis.^7^ One key feature of the tumor microenvironment is hypoxia, which can be generated through increased oxygen consumption in the rapidly proliferating tumor cells, coupled with poor oxygen diffusion through the dense stromal tissue and poor vascularization.^8,9^ The hypoxic microenvironment in PDAC is related to tumor progression, and is described as one of the independent prognostic factors for PDAC.^10^ The adaptive response to hypoxia, primarily mediated by hypoxia-inducible factors (HIFs), confers a more aggressive and therapeutically resistant phenotype in PDAC cells.^9^ Hypoxia promotes the stabilization and activation of HIFs, which regulate the expression of numerous genes involved in angiogenesis, metabolism, cell survival, and invasion.^11^ HIF-1α, a transcription factor, plays a significant role in cellular proliferation, apoptosis, and angiogenesis under hypoxic conditions and could be used as a therapeutic target.^12,13^ Therefore, understanding the mechanisms by which hypoxia influences PDAC biology is crucial for developing targeted therapies aimed at mitigating hypoxia-driven tumor progression and improving patient outcomes.

microRNAs (miRNAs/miRs) are small noncoding RNA molecules, typically 21–25 nucleotides long, that play crucial roles regulating gene expression by binding to mRNA, and affecting cellular proliferation, differentiation, and apoptosis.^14^ Based on their impact on downstream signaling pathways and disease progression, miRs can be classified as either oncogenic (oncomiRs) or tumor suppressive. In the context of PDAC, miRs have emerged as critical modulators of tumor biology, impacting tumor initiation, progression, and metastasis, which makes them novel biomarkers and potential therapeutic targets.^15^ miR-210 is one of the most common oncomiRs in PDAC; high levels of miR-210 found in PDAC tissues and plasma are a predictor of poor outcome.^16,17^ Under hypoxic conditions, miR-210 is overexpressed in response to HIFs, leading to cellular alterations, including cell cycle regulation, mitochondria function, apoptosis, angiogenesis, and metastasis.^18,19^ Furthermore, miR-210 can be used as a predictive marker for tumor hypoxia, as the expression levels of miR-210 are dependent on the level of HIF1-α. HIFs are primary regulators of cellular reactions to oxygen deprivation.^20^

Hypoxic areas within tumors maintain stem-like properties of cancer cells, which promotes self-renewal and metastatic ability.^21^ Cancer stem cells (CSCs) play a vital role in chemoresistance and the metastasis of numerous malignancies, including PDAC.^22^ Specifically, pancreatic CSCs (PCSCs), such as CD44, CD24 and CD133, are less than 1% of all pancreatic cancer cells and act as regulators of PDAC tumor growth, maintenance, metastasis, and chemoresistance.^23^ CD133 expression has been linked to progenitor/stem cells, tumors, regeneration, differentiation, and metabolism, while hypoxia-induced CD133 expression is also found in pancreatic cancer cells.^24,25^ CD44, a cell surface adhesion receptor, is found to be dysregulated in several malignancies and regulates metastasis via the recruitment of CD44 to the cell surface.^26^ CD24 is a highly glycosylated cell adhesion protein, which plays an essential role in tumorigenesis and represents a target of HIF1-α.^27^

Epithelial–mesenchymal transition (EMT) is a key process in the metastatic cascade, characterized by enhanced cell motility, suppression of E-cadherin, and upregulation of mesenchymal markers like Vimentin and Snail.^28^ For example, CD133 facilitated cell migration and promotes EMT through the increased expression activity of HIF1-α under hypoxia.^29^ Moreover, hypoxia or upregulation of HIF1-α leads to EMT in PDAC cells, which is closely associated with NF-κB activity.^30^ Activation of the HIF1-α-Snail regulatory axis promotes EMT in hypoxic PDAC cells.^31^ Further studies have also shown the role of hypoxia-induced miR-210 in EMT, angiogenesis and endothelial cell permeability via exosomes in PDAC.^15,30,32^

Our study investigated the role of miR-210 in PDAC cell lines Panc-1 and MiaPaCa-2 under both normoxic and hypoxic conditions. Hypoxic exposure induced stemness by increasing CD24, CD44, and CD133 mRNA levels. E-cadherin mRNA decreased while upregulating Vimentin; *Snail* gene expressions were further upregulated. Depletion of miR-210, an antagonist of HIF1-α, into hypoxic Panc-1 and MiaPaCa-2 cells reversed the EMT and CSC marker expression levels. Therefore, our results confirm that miR-210 plays a pivotal role in modulating hypoxic responses in PDAC, suggesting potential avenues for targeted therapeutic strategies.

## Results

### miR-210 is Upregulated in PDAC Patient Samples

**Figure 1:**
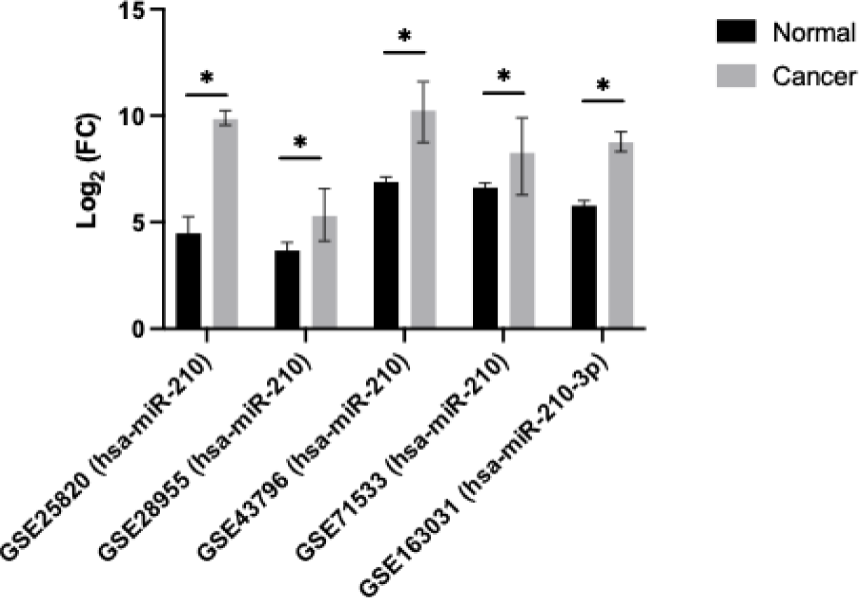
miR-210 expression is upregulated in PDAC patient tissue samples. The expression profile of miR-210 is compared between normal pancreatic tissue samples (or pancreatitis tissue) and PDAC tissue samples. The expression levels of miR-210 are derived from data in 5 previous studies available in the GEO database. Differential expression of miR-210 had been normalized by log2, and adjusted *p* < 0.05; error bars indicate standard deviation (SD).

RNA-seq and miRNA-seq data sets from the Gene Omnibus (GEO) and the Cancer Genome Atlas (TCGA) databases show that miR-210 is upregulated in patient PDAC tissue samples (Figure 1). Expression analysis shows that 5 enlisted previous transcriptomic studies on PDAC indicated a significantly increased likelihood of developing PDAC in high miR-210 expression profile versus low miR-210 expression profile.

### Development of miR-210 KO Panc-1 and MiaPaca-2 Cell Lines

**Figure 2:**
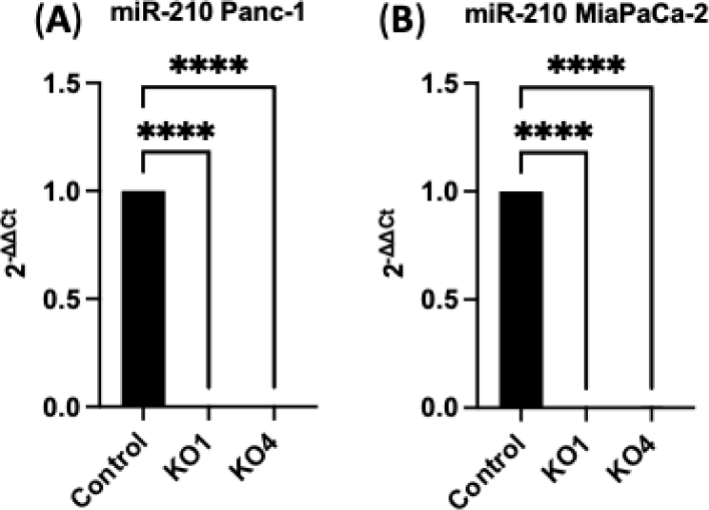
Downregulation of miR-210 expression levels in Panc-1 and MiaPaCa-2 KOs. (A) miR-210 expression in miR-210 KO Panc-1 cells. Expression levels of miR-210 were remarkably downregulated in both miR-210 KO1 and miR-210 KO4 cells compared to control Panc-1 cells (*n* = 3; *p* < 0.0001 for all). (B) miR-210 expression in miR-210 KO MiaPaCa-2 cells. Panc-1, miR-210 expression levels in miR-210 KO1 and miR-210 KO4 cells were significantly reduced compared to control MiaPaCa-2 cells (*n* = 3; *p* < 0.0001 for all). The bar graphs represent the mean of three RNA replicates isolated from control and miR-210 KOs Panc-1 and MiaPaCa-2 cells. Data analyzed using one-way ANOVA followed by Dunnett’s test. Data normalized according to RNU6 expression and analyzed using fold analysis (*n* = 3, *p* < 0.05). *p*-values are indicated as follows: **** *p* ≤ 0.0001.

Panc-1 and MiaPaCa-2 cells express similar levels of miR-210 (data not shown). Expression analysis showed that miR-210 was significantly reduced 99-fold, both in Panc-1 knockout 1 (KO1) and knockout 4 (KO4) compared with the Panc-1 control (*n* = 3; *p* < 0.0001 for all; Figure 2A). Similarly, expression levels of miR-210 were also significantly decreased down to virtually undetectable levels, both in KO1 and in KO4 (*n* = 3; *p* < 0.0001 for all; Figure 2B) in MiaPaCa-2 cells. The knockout miR-210 Panc-1 and MiaPaCa-2 PDAC cell lines were further assessed for the EMT and CSC markers.

### HIF1-α Expression Levels under Hypoxic Conditions

**Figure 3:**
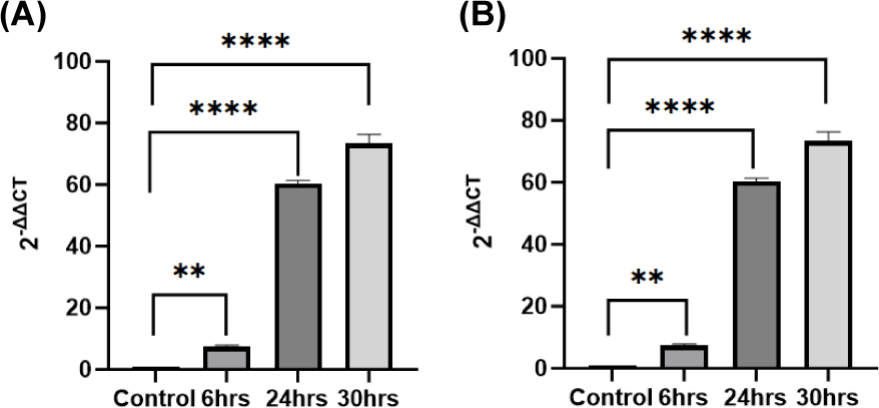
HIF1-α expression levels in (A) Panc-1 and (B) MiaPaCa-2 cells exposed to hypoxic conditions for 6, 24, and 30 h by using RT-qPCR. The 24 h hypoxic exposure was selected for CSC and EMT markers experiments. Data represent the mean ± SD of three technical replicated, analyzed using one-way ANOVA followed by Dunnett’s test. Data normalized according to RPII expression and analyzed using fold analysis (*n* = 3, *p* < 0.05). *p*-values are indicated as follows: ** *p* ≤ 0.01; **** *p* ≤ 0.0001; error bars indicate SD).

HIF1-α relative expression levels were examined under normoxia and hypoxia conditions in different time points (6, 24, and 30 h) by using RT-qPCR. In Panc-1 cells, HIF1-α expression levels showed a significant increase, rising 7-fold at 6 h (*n* = 3, *p* < 0.01), 60-fold at 24 h (*n* = 3; *p* < 0.0001), and 75-fold at 30 h (*n* = 3; *p* < 0.0001) (Figure 3A). Similarly, in MiaPaCa-2 cells, HIF1-α expression levels were elevated 5-fold at 6 h (*n* = 3, *p* < 0.01), and by 60-fold at 24 h and by 80-fold at 30 h (*n* = 3; *p* < 0.0001 for both) (Figure 3B). Based on this data, we chose the 24-h hypoxic exposure and assessed the CSC and EMT markers and 6 h for miRNOME.

**Figure 4:**
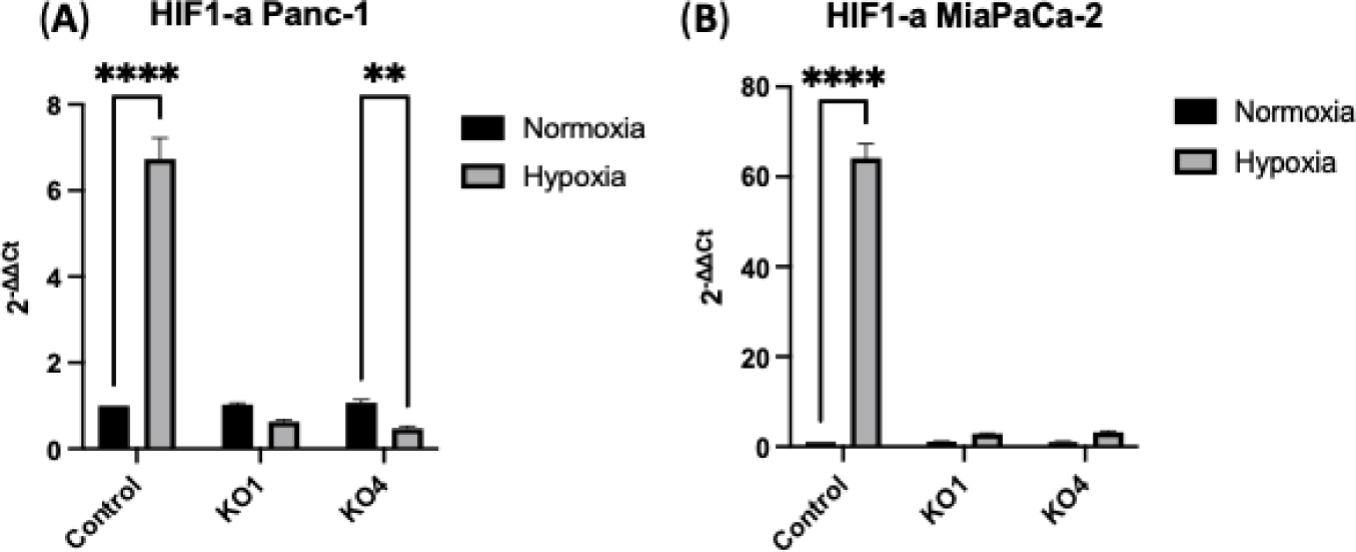
HIF1-α expression levels in (A) Panc-1 and (B) MiaPaCa-2 cells along with their miR-210 knockouts (KO1, KO4) under both normoxic and hypoxic conditions (24 h). HIF1-a expression levels were significantly upregulated in both Panc-1 and MiaPaCa-2 hypoxic control cells compared to normoxia (*n* = 3, *p* ≤ 0.0001). Data represent the mean ± SD of three technical replicated, analyzed using two-way ANOVA followed by Šídák’s test. Data normalized according to RPII expression and analyzed using fold analysis (*n* = 3, *p* < 0.05). *p*-values are indicated as follows: ** *p* ≤ 0.01; **** *p* ≤ 0.0001; error bars indicate SD).

When examining HIF1-α levels under normoxia and hypoxia (24 h) in both Panc-1 and MiaPaCa-2 cells and their miR-210 KO clones, we observed crucial differences. In Panc-1 control cells, HIF1-α expression levels were significantly increased by 6-fold (*n* = 3; *p* < 0.0001) under hypoxia, whereas in Panc-1 miR-210 KO4 cells, HIF1-α levels were significantly decreased by 0.6-fold (*n* = 3; *p* < 0.01) under the same conditions. There were no significant differences in HIF1-α expression between normoxia and hypoxia of Panc-1 miR-210 KO1 cells (Figure 4A). Similarly, in MiaPaCa-2 control cells, HIF1-α expression levels were significantly elevated by 64-fold (*n* = 3; *p* < 0.0001) under hypoxia. No significant differences were observed in the expression levels of HIF1-α between normoxia and hypoxia in MiaPaCa-2 miR-210 KO1 and KO4 cells (Figure 4B). This data underscore the critical role of miR-210 in regulating HIF1-α expression under hypoxic conditions, highlighting a key difference between controls and miR-210 KOs.

### miR-210 KOs Underscores a Significant Impact of PDAC Cells Stem-Like Properties

**Figure 5:**
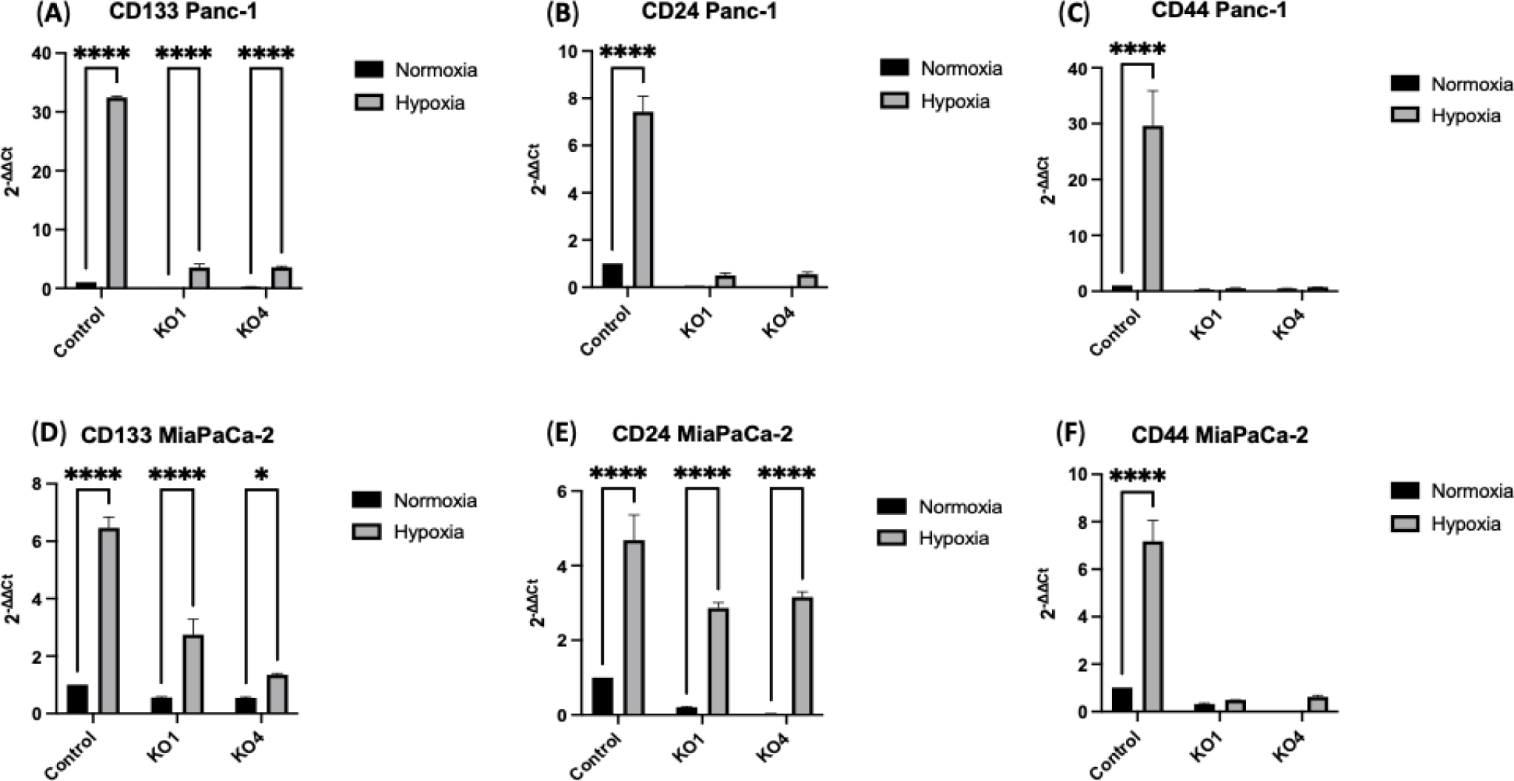
Alteration of CSCs marker expression levels in PDAC cell lines following miR-210 knockout under normoxic and hypoxic conditions (24 h). (A) CD133, (B) CD24, and (C) CD44 mRNA expression in the Panc-1 miR-210 KOs cells, compared with the Panc-1 control. (D) CD133, (E) CD24, and (F) CD44 mRNA expression in the MiaPaCa-2 miR-210 KOs cells, compared with the MiaPaCa-2 control. This figure illustrates the impact of miR-210 depletion on the expression of key CSCs markers in PDAC cell lines. The bar graphs represent the mean of three RNA replicates isolated from control and miR-210 KO Panc-1 and MiaPaCa-2 cells. Data analyzed using two-way ANOVA followed by Šídák’s test. Data normalized according to RPII expression and analyzed using fold analysis (*n* = 3, *p* < 0.05). *p*-values are indicated as follows: * *p* ≤ 0.05; **** *p* ≤ 0.0001; error bars indicate SD.

The investigation of CSCs marker expression levels in both Panc-1 and MiaPaCa-2 PDAC cell lines, as well as their miR-210 KOs under normoxic and hypoxic conditions (24 h), revealed crucial findings. In Panc-1 control cells, CD133 expression levels were significantly increased by 32-fold (*n* = 3; *p* < 0.0001) under hypoxia compared to Panc-1 control cells under normoxia. Similarly, CD133 expression levels were significantly elevated by 3.5-fold (*n* = 3; *p* < 0.0001) in both Panc-1 miR-210 KO1 and KO4 under hypoxia compared to normoxia, highlighting a significant role of miR-210 KOs (Figure 5A). Similarly, CD24 expression levels were significantly increased by 7-fold (*n* = 3; *p* < 0.0001) in Panc-1 control cells compared to Panc-1 control cells under normoxia. No significant differences were observed in the expression levels of CD24 in Panc-1 miR-210 KO1 and KO4 under hypoxia or normoxia (Figure 5B). Furthermore, CD44 expression levels were significantly increased by 30-fold (*n* = 3; *p* < 0.0001) in Panc-1 control cells under hypoxia, yet no significant differences were observed in the expression levels of CD44 in Panc-1 miR-210 KO1 and KO4 under hypoxia and normoxia (Figure 5C).

In MiaPaCa-2 PDAC cells, CD133 expression levels were significantly increased under hypoxia by 6-fold (*n* = 3; *p* < 0.0001), 3-fold (*n* = 3; *p* < 0.0001), and 1.35-fold (*n* = 3; *p* < 0.05) in MiaPaCa-2 control cells, miR-210 KO1, and miR-210 KO4, respectively, compared to normoxia (Figure 5D). Moreover, CD24 expression levels were significantly elevated 5-fold, 2.85-fold, and 3.15-fold (*n* = 3; *p* < 0.0001, for all) in MiaPaCa-2 control cells, miR-210 KO1, and miR-210 KO4, respectively, under hypoxia, compared to normoxia (Figure 5E). CD44 expression levels were significantly elevated by 7-fold (*n* = 3; *p* < 0.0001) in MiaPaCa-2 control cells under hypoxia compared to MiaPaCa-2 control cells under normoxia. No significant differences were observed in the expression levels of CD44 in MiaPaCa-2 miR-210 KO1 and KO4 under hypoxia and normoxia (Figure 5E). The marked reduction in the expression of CSC markers in the miR-210 KOs cells emphasizes the critical role of miR-210 in regulating these markers, which may have important implications for PDAC progression and treatment.

### miR-210 KOs Alter Expression Levels of EMT-Related Markers in PDAC

**Figure 6:**
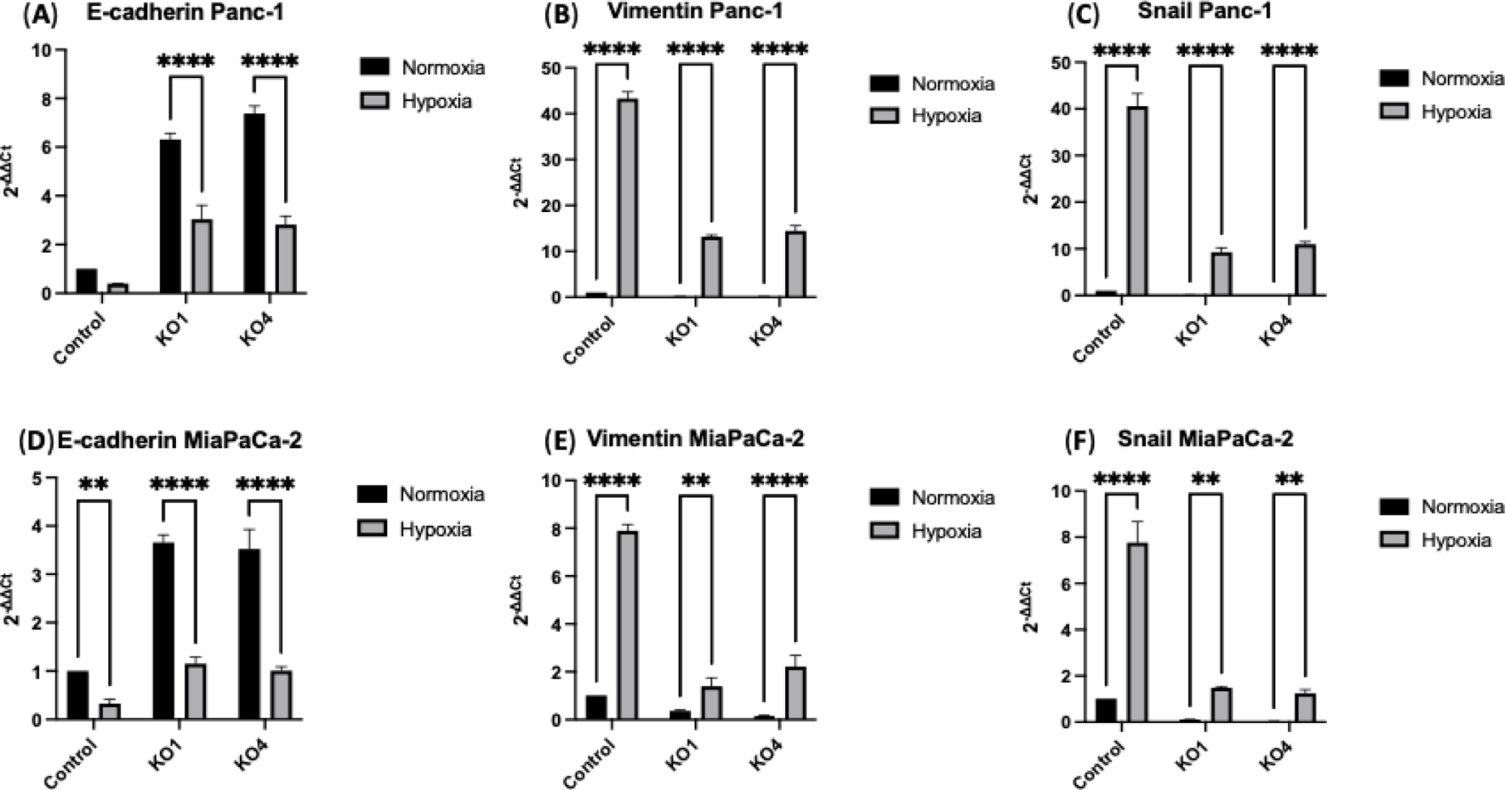
Expression of EMT markers in PDAC cell lines following miR-210 knockout under normoxia and hypoxia. (A) E-cadherin, (B) Vimentin, and (C) Snail mRNA expression levels in the Panc-1 control cells and its miR-210 KOs cells. (D) E-cadherin, (E) Vimentin, and (F) Snail mRNA expression levels in the MiaPaCa-2 control cells and its miR-210 KOs cells. This figure demonstrates the changes in the expression of EMT markers in PDAC cell lines after miR-210 knockout, evaluated under normoxic and hypoxic conditions. The bar graphs represent the mean of three RNA replicates isolated from control and miR-10 KO Panc-1 and MiaPaCa-2 cells. Data analyzed using two-way ANOVA followed by Šídák’s test. Data normalized according to RPII expression and analyzed using fold analysis (*n* = 3, *p* < 0.05). p-values are indicated as follows: ** *p* ≤ 0.01; **** *p* ≤ 0.0001; error bars indicate SD.

The impact of miR-210 knockout on key EMT regulation was evaluated in Panc-1 and MiaPaCa-2 PDAC cells and their KOs under both normoxic and hypoxic conditions. In Panc-1 miR-210 KO1 cells, E-cadherin mRNA expression levels were significantly reduced by 3-fold under hypoxia compared to normoxia, while in miR-210 KO4 cells decreased by 2.81-fold under hypoxia (*n* = 3; *p* < 0.0001, for all, Figure 6A). No significant difference was observed in the expression levels of E-cadherin in Panc-1 control cells under normoxia and hypoxia. Vimentin mRNA expression levels were significantly increased by 43.32-fold in Panc-1 control cells, by 13.17-fold in Panc-1 miR-210 KO1, and by 14.42-fold in Panc-1 miR-210 KO4 under hypoxia (*n* = 3; *p* < 0.0001, for all, Figure 6B). Similarly, Snail mRNA expression levels were significantly elevated by 40.56-fold in Panc-1 control cells, by 9.26-fold in Panc-1 miR-210 KO1, and by 11-fold in Panc-1 miR-210 KO4 under hypoxia (*n* = 3; *p* < 0.0001, for all, Figure 6C).

In MiaPaCa-2 cells, E-cadherin mRNA expression levels were found to be significantly downregulated by 0.33-fold in MiaPaCa-2 control cells (*n* = 3; *p* < 0.01), by 1.15-fold in miR-210 KO1 and by 1-fold in miR-210 KO4 under hypoxia in comparison to normoxia (*n* = 3; *p* < 0.0001 for both) (Figure 6D). Vimentin mRNA expression levels were significantly increased by 7.9-fold in MiaPaCa-2 control cells (*n* = 3; *p* < 0.0001), by 1.4-fold in MiaPaCa-2 miR-210 KO1 (*n* = 3; *p* < 0.01), and by 2.22-fold in MiaPaCa-2 miR-210 KO4 (*n* = 3; *p* < 0.0001) under hypoxia (Figure 6E). Snail mRNA expression levels were significantly elevated by 7.76-fold in MiaPaCa-2 control cells (*n* = 3; *p* < 0.0001), by 1.48-fold in MiaPaCa-2 miR-210 KO1, and by 1.25-fold in MiaPaCa-2 miR-210 KO4 (*n* = 3; *p* < 0.01 for both) under hypoxia (Figure 6F).

### miRNOME of PDAC Cells under Hypoxic Conditions

**Figure 7:**
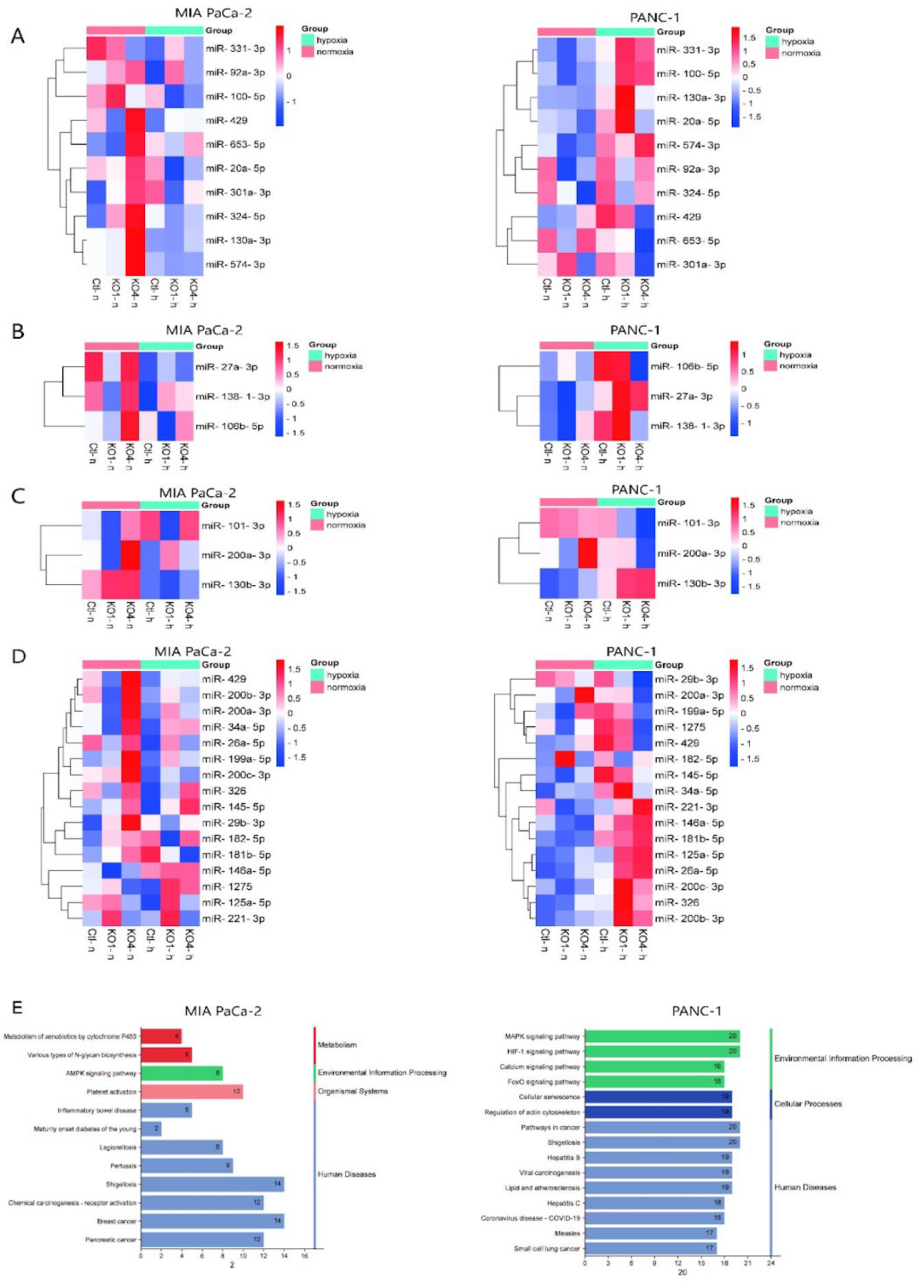
Small RNA-seq of Panc-1 and MiaPaCa-2 miR-210 KO or control cells under normoxia or hypoxia treatment. miRBase miR enrichment of (A) Cellular response to hypoxia (Reactome, experimental (strong)), (B) HIF1-α signaling pathway (GO-biological process), (C) hypoxia-mediated EMT and Stemness (WikiPathways), and (D) response to hypoxia (GO-biological process). (E) GSEA results of MiaPaca-2 and Panc-1 control cells under normoxia and hypoxia were obtained with KEGG pathway annotations.

To investigate the downstream effects of miR-210 KOs under normoxic or hypoxic conditions, small RNA-seq was performed to examine the miRNOME for Panc-1 and MiaPaCa-2 miR-210 KOs to compare to the control cells. Gene ontology (GO) enrichment analysis (miRBase annotation) reveals that in MiaPaCa-2 cells, miRs enriched to the cellular response to hypoxia and HIF1-α signaling pathway are downregulated upon miR-210 KOs under hypoxic conditions. In contrast, opposite expressional changes were observed in Panc-1 cells (Figure 7A,B). miRs enriched to hypoxia-mediated EMT and stemness (Wikipathways) and response to hypoxia (GO-biological process) displayed downregulation upon miR-210 KO under hypoxic conditions in MiaPaCa-2 cells, while those upregulated in Panc-1 cells were similar to the previous two enrichment results (Figure 7C,D). These opposite expressional changes between MiaPaCa-2 and Panc-1 cells under miR-210 KO and hypoxic conditions suggested that miR-210 was correlated with HIF1-α, EMT, and CSC-related miRs and pathways, while the downstream effects were various among different pancreatic cancer cell lines. miRNA gene set enrichment analysis (GSEA) by KEGG pathway annotations indicated that miR changes of Panc-1 and MiaPaCa-2 cells under hypoxic conditions were enriched to the HIF1-α pathway, MAPK pathway, and pathways in cancer. Notably, miR-210 expression landscapes of MiaPaCa-2 cells were enriched to pancreatic cancer under hypoxic conditions. (Figure 7E). This result suggests that PDAC cells respond to hypoxia through miR-210-mediated activation of the HIF1-α pathway, increasing the level of tumorigenesis and PDAC progression.

## Discussion

PDAC exhibits higher levels of hypoxia compared to most solid tumors and intratumoral hypoxia is linked to a poor prognosis in PDAC patients.^33^ Specifically, the oxygen (O_2_) pressure is between 30–50 mmHg in normal tissues, while the pressure is reduced to below 2.5 mmHg in up to 50–60% of locally advanced solid tumors.^34^ HIFs, the key transcription factors regulating adaptive responses to changes in tissue oxygenation, play a significant role in various hypoxia-induced malignant characteristics of PDAC. These characteristics are closely interrelated, forming a signaling network within the hypoxic microenvironment of PDAC. In this study, we investigated the role of miR-210, a hypoxia regulator miR, at the molecular level, specifically examining its impact on EMT and CSC gene expression levels following hypoxic exposure.

Hypoxia is a common condition of the tumor microenvironment. Emerging data suggest that a hypoxic microenvironment might play a pivotal role in the progression of solid tumors. CD133 is a pentaspan transmembrane glycoprotein, which is used as a biological marker for stem cells and CSCs.^35^ Furthermore, CD133+ cells colocalize to the hypoxic areas within pancreatic cancer, further leading to elevated HIF-1α activity.^36^ CD133 is considered a marker of metastatic phenotype through the overexpression of *N*-cadherin via the Src signaling pathway, which plays a vital role in the EMT regulatory loop.^37^ CD24, a small cell surface protein anchored by glycosylphosphatidylinositol, is highly glycosylated and plays a role in cell–cell and cell–matrix interactions. It is generally expressed at elevated levels in progenitor and metabolically active cells, while its expression is lower in well-differentiated cells.^38^ CD44 is a transmembrane glycoprotein that serves as a receptor for extracellular matrix components such as hyaluronic acid and acts as a downstream target of the Wnt/β-catenin pathway. Its expression is associated with more aggressive disease progression and metastasis in PDAC.^39^ Immervoll et al. (2011) previously investigated the expression of CD44 and CD133 in surgical samples from PDAC, noncarcinoid pancreatic tumors, and healthy pancreas tissue using immunohistochemistry and immunofluorescence. Their results suggested the presence of CD44 and CD133 expression in both normal pancreatic tissue and inflammatory pancreatic tumors.^40^ In another study, CD44 levels were assessed in the serum of patients undergoing chemotherapy for PDAC, colon cancer, and gastric cancer, finding that CD44 levels decreased in patients who responded to treatment.^41^ In our study, we demonstrated that depletion of miR-210 in Panc-1 and MiaPaCa-2 cells using CRISPR-Cas9 led to a significant downregulation of mRNA expression levels of CSC markers including CD133, CD24, and CD44 depending on miR-210 status.

Oxygen concentration in the microenvironment acts as a dynamic regulator of cellular plasticity. Intratumorally hypoxia triggers EMT in PDAC, while exposure to normoxia or hyperoxia can reverse EMT. Several studies have highlighted the critical roles of HIF1-α and NF-κB in hypoxia-induced EMT.^42,43^ EMT is a crucial pathway regulating the CSC phenotype. Specifically, CSC populations present increased expression levels of EMT transcription factors, such as Snail, and mesenchymal markers, such as Vimentin, and reduced E-cadherin compared to non-CSC populations.^44^ E-cadherin, a well-known EMT marker, is a calcium-dependent adhesion molecule with leading roles in cell growth, differentiation and apoptosis.^45^ A recent study by Wang et al. (2018) showed that E-cadherin mRNA expression levels were reduced by microenvironmental changes, such as hypoxia, in pancreatic cancer and induced EMT.^46^ Vimentin is expressed in normal mesenchymal cells, and its key roles are to maintain cellular integrity and promote resistance to stress.^47^ Recent studies have also demonstrated that the mRNA expression levels of Vimentin have been increased under hypoxia, which has induced cellular migration and invasion of PDAC cells.^46,48^ Snail, a repressor of E-cadherin expression, has been associated with elevated mesenchymal marker expression, decreased expression of several epithelial markers, inhibition of proliferation and promotion of apoptosis.^49^ In PDAC, Snail expression levels are overexpressed and linked to invasion and metastasis.^50^ Moreover, it has been suggested that Snail expression can be induced by hypoxia, which can be further moderated by HIF1-α expression at the transcriptional level.^51,52^ Our current study showed that miR-210 regulates the stemness of PDAC cells and cellular viability and seems to play an essential role in EMT pathways. Notably, miR-210 KOs reversed the expression levels of E-cadherin, Vimentin, and Snail. Previously, hypoxia was indicated to trigger tumor cells to undergo EMT. However, the exact mechanisms remain unclear. It has been demonstrated that hypoxia-induced dimerization of HIF1-α and HIF1-β within nuclei plays a crucial role in tumor metastasis. The HIF1-α and HIF1-β heterodimer bind to the hypoxia-response element (HRE) and activate EMT-related genes, thereby enhancing the invasive potential of cancer cells.^53^

miR-210 is the most consistently and significantly induced miR during hypoxia, and it is unique in being induced in almost all cell lines.^54^ miR-210 expression regulates both HIF1-α and HIF2-α.^55^ Previous research indicates that cellular and exosomal miR-210 expression is upregulated in PDAC following gemcitabine treatment, suggesting that hypoxia-mediated signaling, which further induces miR-210, could be a critical regulatory factor for PDAC prognosis.^56^ Notably, miR-210 is upregulated by HIF1-α and, in turn, negatively regulates HIF1-α transcript and protein levels in peripheral T cells.^57^ Moreover, miR-210 expression levels hold significant promise as predictive biomarkers for identifying patients who are more likely to benefit from therapies targeting hypoxic pathways. Due to its upregulation under hypoxic conditions and its role in promoting tumor progression and treatment resistance in PDAC, miR-210 can be leveraged to stratify patients based on their tumor’s hypoxic profile.^58,59^ Our data also confirm that miR-210 is upregulated by hypoxia and correlates with HIF1-α expression (Figure 4). Additionally, it has been confirmed that miR-210 is a direct target of HIF3-α.^60^ miR-210 could act as a switch between HIF-1/2 and HIF3-α to inhibit HIF3-α under normal conditions. During chronic hypoxia, however, HIF-1/2 levels may no longer be controlled by HIF3-α, leading to accumulation of HIF3-a. Furthermore, miR-210 has emerged as a promising target for therapeutic interventions aimed at mitigating hypoxia-driven tumor progression in PDAC. Given its critical role in regulating cellular adaptation to hypoxic conditions, targeting miR-210 could disrupt the hypoxia-induced pathways that contribute to tumor aggressiveness, treatment resistance, and poor prognosis. Therapeutic approaches, such as miR-210 inhibitors or miRNA-modulating agents, may help reduce the hypoxic response in tumor cells, improving the efficacy of existing chemotherapeutic and radiotherapeutic strategies.^61−63^ The use of miR-210 as a biomarker could thus enhance personalized treatment approaches, leading to better therapeutic outcomes by tailoring treatment plans to the hypoxic status of the tumor. Therefore, in our study, we investigated the role of miR-210 by removing miR-210 from PDAC cell lines via the HIF1-α axis. Further research is required to understand the mechanistic role of miR-210 on HIF3-α accumulation during chronic hypoxic conditions such as PDAC.

Small RNA-seq results reveal a landscape of downstream effects of miR-210 knockout and hypoxic conditions. Our study mainly focuses on the miRs that functionally enrich cellular response to hypoxia, HIF1-α pathways, hypoxia-mediated EMT and stemness, and biological processes of hypoxia responses. Our results unexpectedly show different expressional changes between two PDAC cell lines upon miR-210 knockout and hypoxic conditions. Moreover, GSEA results indicate that miR-210-mediated activation of cellular hypoxic responses correlates with the cancer pathways. These expressional changes in candidate miRs hint that miR-210 regulates HIF1-α, CSC, and EMT in a sophisticated manner, which requires future research of target gene analysis. Our results indicate the necessity of an in vivo study to demonstrate how miR-210 knockdown can affect the PDAC tumor. miRs of the hypoxia-induced HIF1-α pathways (miR-138-1-3p, miR-106-5p, and miR-27a-3p) were reported to be correlated with the stemness, metastasis, and progression of nasopharyngeal carcinoma, esophageal squamous cell carcinoma, and esophageal cancer.^64−66^ miR-210 knockout PDAC cells under hypoxic conditions displayed differential expression in the miRs under hypoxia-induced HIF1-α pathways, which suggested correlations among miR-210, cancer stemness, and metastasis. GO enrichment of the cellular response to hypoxia showed alterations in the expression of candidate miRs, indicating that other hypoxia-related pathways were targeted by miR-210. miR-101-3p, miR-200a-3p, and miR-130b-3p were enriched to hypoxia-induced stemness and EMT. Previous studies reported that miR-130b-3p targeted the EphB4/JAK2/STAT3 axis to reduce the stemness of pancreatic cancer under the treatment of Erlotinib and Gemcitabine.^67^ GO enrichment analysis provided a set of miRs potentially targeted by miR-210 under hypoxic conditions in PDAC. Under hypoxic conditions, GSEA analysis of miR-210 knockout Panc-1 cells showed enrichment of HIF-1, MAPK, calcium, and FoxO signaling pathways with small-cell lung cancer. In miR-210 knockout MiaPaCa-2 cells, the AMPK signaling pathway was enriched with pancreatic and breast cancer. Enrichment results indicated a correlation among hypoxia-induced miR-210, PDAC progression, and proinflammatory signaling pathways, which requires further research to elucidate the cellular responses. In this study, our primary focus was on the fine-tuning effects of miRs at the mRNA level. Although mRNA expression does not always correlate with protein abundance due to post-transcriptional regulatory mechanisms, protein expression data provide a more comprehensive understanding of the downstream effects of miR regulation. Future studies will aim to address this by incorporating proteomic analyses. HIF1-α has been identified as a potential therapeutic target for various diseases, including cancer.^68^

**Figure 8:**
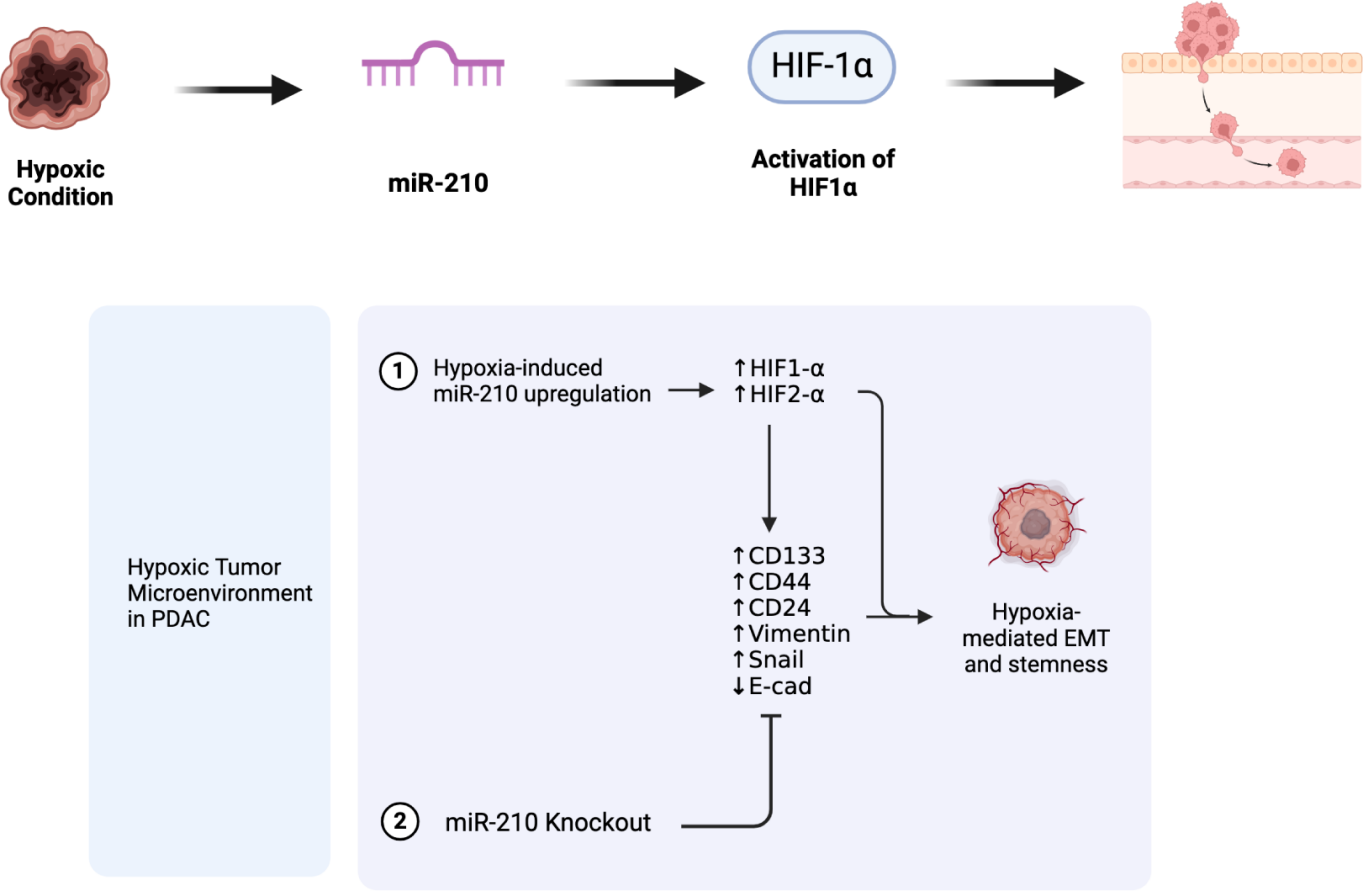
Role of miR-210 in hypoxia-induced PDAC progression. This figure illustrates the functional role of miR-210 in promoting PDAC progression under hypoxic conditions. miR-210 influences the expression of key EMT and CSCs markers involved in hypoxia signaling pathways linked to tumor progression. Created in BioRender. Mortoglou, M. (2024) https://BioRender.com/ b53u347.

Our in vitro studies uniquely show that miR-210 is a potent regulator of HIF1-α, CSCs and EMT under hypoxic conditions (Figure 8). The observed differences in miRNA expression between Panc-1 and MiaPaCa-2 cells under hypoxia likely arise from intrinsic variations in their hypoxic response mechanisms, leading to the differential activation of hypoxia-responsive miRs, such as miR-210. These cell-line-specific differences in HIF1-α signaling pathways, metabolic adaptations, and transcriptional profiles could account for the disparate miRNA responses observed under hypoxic conditions. Understanding these mechanisms requires further investigation into how each cell line uniquely modulates hypoxia-induced miRNA expression. Our study offers detailed insights into these molecular mechanisms, laying the groundwork for novel therapeutic strategies aimed at targeting hypoxia-induced signaling via miR-210 modulation and providing a basis for future clinical investigations. Consequently, targeting HIF1-α by administering miR-210 mimics in vivo may represent an effective therapeutic strategy for PDAC.

## Conclusions

PDAC presents higher levels of hypoxia than most solid tumors, whereas HIFs are closely associated with several hypoxia-induced malignant phenotypes of PDAC. Together, the findings presented in this study highlight the central role of miR-210 in HIF signaling, cancer stemness, and EMT pathways under normoxic and hypoxic conditions. miR-210 KOs reversed the mRNA expression levels of the EMT and CSCs markers. miR-210 knockout and hypoxic conditions suggested that miR-210 was correlated with HIF1-α, EMT, and CSC-related pathways. However, the molecular mechanisms that correlate hypoxic signals to EMT, cancer stemness, and metastasis should be further examined.

## Methodology

### miR-210 Expression from Public Databases

miR-210 expression level data in human patient tissues were obtained from TCGA-GDC miRNA-seq data sets, consisting of 177 pancreatic cancer tumor samples and 4 normal/pancreatitis samples. miRNA-seq data was downloaded in TPM format and visualized using GraphPad Prism 9 (La Jolla CA, USA) software. Five individual GEO series (GSE25820, GSE28955, GSE43796, GSE71533, and GSE163031) were obtained to further consolidate the expression level of miR-210 in patient tissues.^69−73^ miRNA-seq data was downloaded in counts format and normalized using R/Bioconductor Package, followed by log_2_ (FC) normalization. Normalized data sets were visualized using GraphPad Prism 9 (LA Jolla Ca, USA).

### Cell Culture and Hypoxia Exposure

PDAC cell lines Panc-1 (ATCC CRL-1469) and MiaPaCa-2 (ATCC CRL-x1420) were obtained from ATCC and cultured accordingly. Cells were grown approximately 80% confluence in 75 cm^2^ flasks using complete Dulbecco’s modified Eagle’s medium (DMEM) supplemented with 10% fetal bovine serum (FBS) as previously described.^74^ For normoxic conditions (21% O_2_), cells were maintained at 37 °C in a humidified environment with 5% CO_2_ as the adherent monolayer. Cells were cultured in a hypoxia chamber (Galaxy 48 R, New Brunswick, Eppendorf, Stevenage, U.K.) at 1% O_2_ with the same 37 °C and 5% CO_2_ humidified environment for the hypoxia experiments.

### CRISPR/Cas9 Assay

sgRNAs targeting human miR-210 were designed using E-Crisp and cloned into lentiCRISPRv2 (Addgene#52961, and a gift from Feng Zhang).^75^ Targeting sequences for KO1 and KO4 were 5′GCGCAGTGTGCGGTGGGCAG3′ and 5′GGGGCAGCGCAGTGTGCGGT3′, respectively. Panc-1 and MiaPaCa-2 stable cell lines were generated by lentiviral transduction, as previously described^76^ and selected with 1.4 μg/mL and 2 μg/mL puromycin, respectively. An sgRNA for eGFP was used as a negative control.

### RNA Extraction and RT-qPCR

RNA extraction was performed by using RNAzol RT (Sigma, Hertfordshire, U.K.) from Panc-1 and MiaPaCa-2 and their miR-210 knockouts (stored at −80 °C). The NanoDrop spectrophotometer (Thermo Fisher Scientific, Hemel Hempstead, U.K.) was used to measure the RNA concentration and integrity levels at absorbance wavelengths of 260 and 280 nm. For cDNA synthesis, the miRCURY LNA RT Kit (Qiagen, Manchester, U.K.) was used as previously described.^75,77^ The resulting cDNA from PDAC cell lines was utilized to measure miR-210 expression levels, with RNU6 serving as the reference gene for the normalization. The MystiCq miR-210 qPCR primers obtained from Sigma (Paisley, U.K.) were used with the miRCURY LNA SYBR Green PCR Kit (Qiagen, Manchester, U.K.). The thermocycling conditions were set as follows: 95 °C for 2 min, followed by 95 °C for 10 s and 56 °C for 60 s.^75,77^ qScript cDNA Supermix (Quantabio, Lutterworth, U.K.) was used to evaluate the mRNA expression levels of E-cadherin, Vimentin, Snail, CD24, CD44, and CD133 was assessed using PrecisionPlus qPCR Master Mix (Primer Design, Chandler’s Ford, U.K.) as previously described.^78^ The primers for Snail and E-cadherin, were designed and purchased from Sigma (Paisley, U.K.), Vimentin from Integrated DNA Technologies (IDT) (Leuven, Belgium), and CD133, CD24, and CD44 from Thermo Fisher Scientific (U.K.).^79^ The comparative CT/2^–ΔΔCT^ method was used to determine the relative mRNA expression levels, with RPII serving as the reference gene.^80^

### Small RNA-Sequencing and Analysis

miRNeasy Micro Kit (Qiagen, U.K.) was used to perform RNA extraction following the manufacturer’s instructions. The NanoDrop Spectrophotometer (Thermo Fisher Scientific, Hemel Hempstead, U.K.) was utilized to measure RNA concentration at 260 and 280 nm absorbance. Quality control, library preparation, and Small RNA sequencing were carried out by Novogene (Cambridge, U.K.) using Illumina Sequencing (SE50). NEBNext Multiplex Small RNA Library Prep Set for Illumina kit was utilized for the library preparation. The raw data included 5′ primer contains, no insert tags, oversize insertion, low quality reads, poly A tags, and small tags, which were excluded from the analysis. Galaxy Software version 23.1.rc1 (https://usegalaxy.org/) was used to further analyze the clean reads. miR id was aligned to miRBase version 21 (https://mirbase.org/) hairpin and mature miR id. Differential expression was determined by the ratio of normalized read counts (CPM) and miRs with expression levels below 100 were filtered out. GO enrichment analysis of differentially expressed miRs were performed by miRPath DB v2.0 using miRBase annotation (https://mpd.bioinf.uni-sb.de/., https://www.mirbase.org/) (miRNA). GSEA was performed by miEAA (https://ccb-compute2.cs.uni-saarland.de/mieaa/) with top 20 miRs that were upregulated or downregulated.^81−84^ Reactome, KEGG, and WikiPathways were selected to determine biological functions or pathways enriched with differentially expressed miRs. Heatmaps of candidate miRNAs were plotted by Heatmapper (http://www.heatmapper.ca/) and R ggplot2 package.

### Data Analysis

One-way ANOVA followed by Dunnett’s test was used to analyze the expression levels of miR-210, and HIF1-a, while for EMT and CSCs markers experiments Two-way ANOVA followed by Šídák’s test was used as previously described.^78,79^ GraphPad Prism v10.2.2 (La Jolla, CA, USA) was utilized for statistical analysis; statistical significance was determined using Tukey’s test at *p* ≤ 0.05, and all the results are presented as mean ± SD.
